# Hysteroscopic sterilization of patient with intrauterine device Mirena^®^

**DOI:** 10.1590/S1679-45082013000100019

**Published:** 2013

**Authors:** Daniella De Batista Depes, Ana Maria Gomes Pereira, Salete Yatabe, Reginaldo Guedes Coelho Lopes

**Affiliations:** 1 Hospital do Servidor Público Estadual “Francisco Morato de Oliveira”, São Paulo, SP, Brazil

**Keywords:** Hysteroscopy/methods, Intrauterine devices, Sterilization, tubal/methods, Case reports

## Abstract

Tubal sterilization is the definitive procedure most often used worldwide to control fecundity. Laparoscopic ligature is safe, but invasive and with possible surgical and anesthetic risks. The hysteroscopic approach enables tubal occlusion at outpatient's setting without the need of incisions or anesthesia. A microdevice (Essure^®^) is inserted directly into the tubes and its polyethelene fibers cause obstruction of tubes in about three months. During this period, it is recommended that patients continue the use of a temporary birth control method. Several women use the levonorgestrel-releasing intrauterine system, which is called in the market as Mirena^®^. This report evaluated the possibility of inserting Essure^®^ without remove the intrauterine device; patient tolerance to the procedure was also assessed. The tubal device was successfully placed in the patient without the need to remove Mirena^®^. After three months the intrauterine device was removed with no intercurrent events.

## RESUMO

A esterilização tubária é o procedimento definitivo mais utilizado no mundo para controle da fecundidade. A ligadura laparoscópica é segura, porém invasiva e com possíveis riscos cirúrgicos e anestésicos. A via histeroscópica permite a oclusão tubária em ambiente ambulatorial, sem incisões ou anestesia. Um microdispositivo (Essure^®^) é inserido diretamente no interior das tubas e suas fibras internas de poliéster causam sua obstrução em até 3 meses. Durante esse período, deve ser mantido o método contraceptivo temporário utilizado pela paciente. Várias mulheres utilizam o sistema intrauterino liberador de levonorgestrel, denominado comercialmente Mirena^®^. O objetivo neste estudo foi de avaliar a possibilidade de inserção do Essure^®^ sem a remoção do dispositivo intrauterino e a tolerância da paciente ao procedimento. O dispositivo foi colocado com sucesso em paciente portadora do Mirena^®^ sem necessidade de retirada do mesmo. Após 3 meses o dispositivo intrauterino foi retirado sem intercorrências.

## INTRODUCTION

Tubal sterilization is the definitive procedure most often used worldwide to control fecundity^(1)^. An ideal contraceptive method should be highly efficacy and of minimal complications. A transcervical access would be an effective alternative to transabdominal, therefore eliminating the need for incisions or general anesthesia.

During the 1990s an intratubal microdevice namely the Essure^®^ was manufactured by Conceptus Inc, based on San Carlos, CA, USA. The procedure consists of canalization of tubes using a catheter inserted transcervically during a hysteroscopy.

This microdevice consists of stainless steel metallic structure, self-expanding nitinol coil and polyester (PET) fibers. The fibers are around the inner coil of stainless steel and surrounded by an expanding outer nickel titanium ring that keep the device in the uterotubal junction during time required to stimulate tissue grow^(2)^.

Due to PET fibers placed in the fallopian tubes there is a reaction of surrounding tissue leading to an irreversible tubal occlusion. Over next three months after the procedure the woman must continue to use another highly effective form of birth control. Three months after placement a pelvic radiograph or an ultrasonography could confirm if the device was appropriately placed^(3)^.

An increase in number of women using Mirena^®^ (levonorgestrel intrauterine device [IUD]) and who opted to hysteroscopic tubal sterilization has been seen. Many authors evaluated if IUD presence could affect the success rates of tubal sterilization procedure^(4–7)^. These patients often have contraindication or intolerance to oral contraceptive medicines, therefore, it is ideal to keep their IUD during tubal occlusion^(5)^.

A study by Agostini et al. proposed to patients using IUD, intolerant to pills and who were candidate to hysteroscopic sterilization to keep their device during the procedure. Six patients were included and all insertions were done with a mean time of five minutes. All placements achieved success without complications. Three months after the procedures all IUDs were removed without difficulties^(5)^.

This report evaluated the possibility of hysteroscopic tubal sterilization even in the presence of Mirena^®^ IUD.

## CASE REPORT

A 25-years-old married woman who had two pregnancies and two previous cesarean sections was diagnosed with mitral and tricuspid insufficiency, and pulmonary hypertension in November 2007. She was advised to avoid new pregnancies. In 2009, the patient was admitted in family planning service of Hospital do Servidor Público Estadual “Francisco Morato de Oliveira” (HSPE-FMO) being the permanent contraception using laparoscopy indicated. After preanesthesia assessment the surgery was contraindicated because the patient was at high risk for anesthesia, therefore, the Mirena^®^ IUD placement was indicated.

After approval of hysteroscopic ligation by the National Health Surveillance Agency (ANVISA, acronym in Portuguese), we suggested this contraceptive method to the patient who immediately agreed.

**Figure 1:**
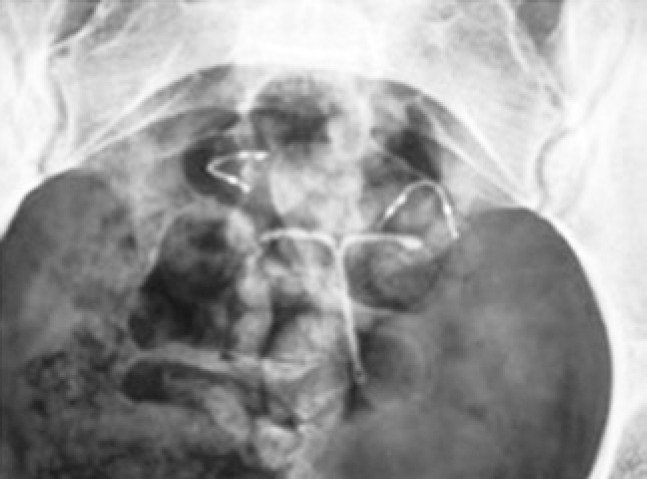
Pelvic x-ray showing intrauterine system and microdevices in tubes

**Figure 2:**
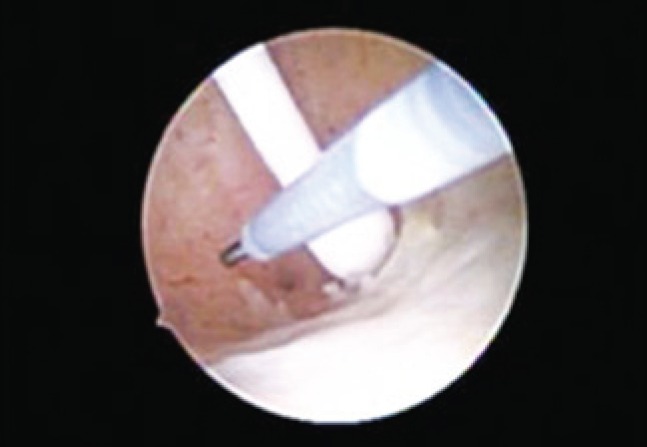
Insertion of microdevices inside ostium of the left tube

**Figura 3:**
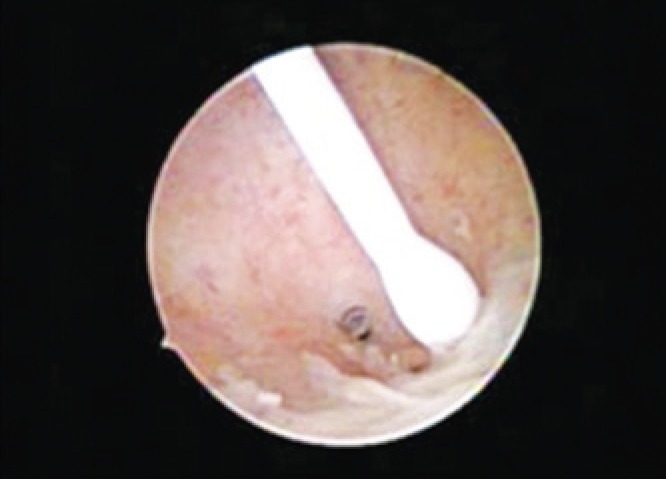
Essure^®^ placed inside ostium of the left tube

The procedure was carried out in the hysteroscopy room of ambulatory gynecology at HSPE-FMO. The technique used was vaginoscopy, as described by Bettocchi and Selvaggi^(8)^, which consists of performing the test without prior digital vaginal examination and without the use of speculum or Pozzi forceps to traction the cervix. A 2.9-mm-scope was used, with a 30° angle, an internal inflow sheath, final diameter of 4mm and oval distal extremity (Bettocchi hysteroscope, Karl Storz^®^, Germany). For vaginal distension, 0.9% saline solution was used, at room temperature, with pressure determined by gravity and pressure cuff filling around the flexible flask, with continuous flow, pressure of approximately 100mmHg, and insufflation sufficient to adequately visualize the cervical canal and uterine cavity. The image was transmitted to a TV monitor, with a 300-W xenon light source. The procedure was carried out with no anesthesia or analgesia. After visualize uterine cavity with Mirena^®^ IUD and tubes, the catheterization and detachment of devices were initiated (Figures 1 to 3). The procedure duration was 5 minutes with no complications.

The patient classified her pain as 3 after the procedure according to analogical pain scale that varied from 0 (no pain) to 10 (worst pain ever experienced). She also did not report any discomfort after the procedure and returned to her daily activities on the same day.

**Figure 4:**
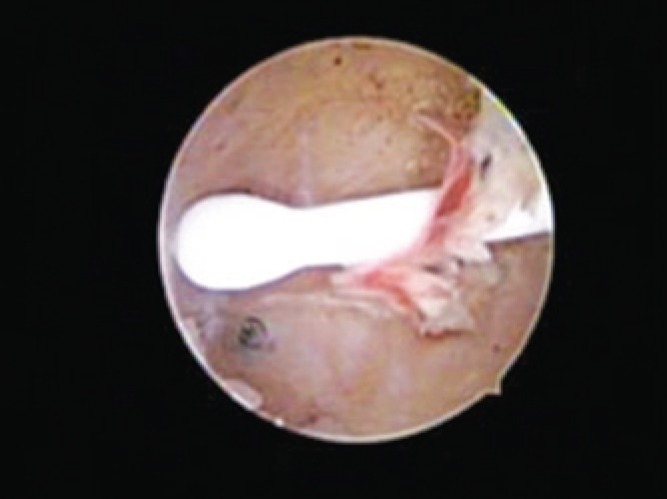
Essure^®^ placed inside ostium of the right tube

Pelvic radiographic after 3 months showed that devices were correctly placed. Subsequently her IUD was removed (Figures 1 to 4).

## DISCUSSION

Hysteroscopic tubal sterilization is a relatively new procedure that may be performed in the ambulatory without anesthesia. It has short learning curve and can be done by any physician with experience in hysteroscopy. The device is easily placed in fallopian tubes, usually with no complications^(5)^.

This method inconvenience is that patients must continue to use other form of birth control during the 3 months required in order to occur tubal occlusion. Women with IUD usually have contraindications or are intolerant to other temporary birth control methods^(5)^.

In 2010, Tatalovich and Anderson^(6)^ showed that hysteroscopic sterilization could be successfully undergone by 12 Mirena^®^ IUD users without the need to remove the device. They also emphasized the fact that the endometrium become quite atrophic in levonorgestrel IUD users which enables to visualize ostium of fallopian tubes and provides contraceptive protection until tubal blockage is confirmed. In addition, they observed that patients with IUD were less likely to be absent on 3 months confirmation test day particularly because they expected to remove the IUD^(6)^.

Sánchez et al.^(7)^ in 2010 assessed results after Essure^®^ placement in women with and without IUD; all procedures were performed in the ambulatory. In the study they observed differences in success rate, level of difficult and patients' tolerance when removed or not the IUD one month later after microdevice insertion. The conclusion was that, even with lower success rates and complications frequently presented by IUD users, insertion was possible in more than 97% of cases.

Although further studies are required to confirm this result, this study showed the feasibility of Essure^®^ intratubal device placement in patients with Mirena^®^ IUD without the need to remove it. Essure^®^ constitutes a good option for patients with contraindication or intolerance to other birth control methods.
